# Influence of Environmental Management on Green Process Innovation: Comparison of Multiple Mediating Effects Based on Routine Replication

**DOI:** 10.3390/ijerph16224346

**Published:** 2019-11-07

**Authors:** Yuan Ma, Qiang Zhang, Qiyue Yin

**Affiliations:** College of Economics and Management, Shandong University of Science and Technology, Qingdao 266590, China; zhangqiang3637@163.com

**Keywords:** environmental management, green process innovation, zero-order routine replication, higher-order routine replication, multiple mediating effects

## Abstract

Although green process technology is vital to sustainable development, few articles focus on how to implement it from the perspective of firms. This article tries to answer this question. Being set as an antecedent of green process innovation, the influence of environmental management is analyzed and the influential path is elaborated. Hypotheses are tested by means of multivariate regression analysis and bootstrap method. The results show that environmental management is conducive to firms’ green process innovation, and the influence is through zero-order routine replication and higher-order routine replication. The mediating effect played by the interaction between the two is stronger than that of the individual. Implications are given to academia and practitioners.

## 1. Introduction

Green process innovation (GPI) is vital to business in order to realize sustainable development [1]. It means the application or exploitation of production processes that is novel to a firm and which results in the reduction of environmental pollution compared to relevant alternatives [1,2,3]. Existing studies have focused on the drivers of GPI from outside firms. Although abundant literature verifies that governmental regulations and stakeholder pressures can trigger the improvement in production process of firms [4,5,6,7,8,9,10,11,12,13], how to develop GPI is still under-researched. Technological innovation cannot happen spontaneously, and innovators often encounter many problems during the innovation process [14,15]. There is an urgent need to address the development of GPI within firms in order to guide firms to operate sustainably [16]. It is the question that this article focuses on.

We try to answer the above question from the perspective of environmental management (EM). EM is the systematic arrangement related to the overall policies, structures, specific responsibilities of employees, and other managerial initiatives firms take to improve environmental performance [17,18,19,20,21]. As a management theory, EM appeared in academia in the 1990s; while, as a managerial practice, it has been adopted and become popular in business in the last 20 years [22,23,24]. The similarity between EM and GPI is that both of them stress the improvement of natural environment. The difference is that the former focuses on managerial initiatives that firms take, and the latter highlights novel technologies that firms get. Innovation theory holds that management practice can provide fertile soil for firms’ technological innovation [25]. So, we posit that EM can facilitate GPI. Although this viewpoint seems apparent, it still remains under-explored. Extant literature mainly focuses on the short-term impact of EM, such as labor productivity [26,27,28], but pays little attention to the long-term impact, such as GPI [22,29]. To fill this research gap, we analyze the under explored EM–GPI relationship.

Process technology in the same industry is always similar, resulting in the same pollutants and emissions [20]. GPI often requires extensive cooperation between firms [1]. Considering the particularity of GPI, routine replication is used as a mediating variable to analyze the EM–GPI relationship in-depth. Routine replication refers to the transfer of routine across firms [30]. Its essence is the transfer of knowledge sets [31,32], which is conducive to technology innovation. We hope this explanation helps deepen understanding of the relationship between EM and GPI.

The research sample in this article is the Chinese manufacturing industry. China is experiencing unprecedented economic and environmental changes [4]. The development model has long placed economic growth above environmental protection, making China one of the most polluted countries [7]. However, the priorities of China’s economic development and environmental protection have begun to change. The government has placed more emphasis on resource conservation and environmental protection [19]. In the 13th Five Year Plan (2016–2020), the Chinese government clearly pointed out that it is vital to gain sustainable development [4,5]. In order to improve natural environment, the central government sends inspection teams periodically. EM is becoming urgent for Chinese firms, but most firms still suspect the impact of EM. So, an interesting phenomenon emerges that many firms halt production to avoid environmental inspections. China provides an interesting context in which to investigate the relationship between EM and GPI.

The potential academic contributions of this paper are as follows: (1) The research enriches extant GPI literature by setting up an EM–GPI framework. GPI is the source of firms’ sustainable competitive advantage. Although extant research around GPI is abundant, there still lacks a guidance of how to develop GPI in firms. Studying the impact of EM on it will contribute to guide firms’ GPI. (2) This paper also investigates the underlying influential mechanism in the EM–GPI relationship by using mediating variables, and compares the possible multiple mediating effects. Many scholars and business practitioners still have doubts about the effect of EM. They hold that it is not the core function of firms, but a decoration or even a burden [33,34]. Through the study of the influential mechanism, the research offers a conceptual framework that could remedy this theoretical deficiency and enhance the confidence of business in EM.

The following contents are arranged as follows. In the second part, the relevant theories are given and hypotheses are proposed. The third part introduces the methodology and the fourth part the analysis results. Finally, implications are given.

## 2. Theoretical Background and Hypotheses

### 2.1. Theoretical Background

EM is a series of environmental policies and management practices that firms adopt in order to reduce, eliminate, and prevent the negative environmental impacts of their operations [17,18,19,35,36]. The typical EM practices are: Setting environmental goals in the organization, adjusting organizational structures, monitoring and auditing environmental performance, and training employees [19,22,33,37,38,39,40,41,42,43]. The goal of these practices is to solve firms’ environmental issues. The short-term impact of EM has been argued for a long time in academia [22,23,24,25,26,27,28], but the long-term benefit, such as GPI, is still underestimated, which affects the adoption of EM in firms [22].

GPI is the exploration and adaption of production process in order to reduce environmental impact of firms [1,2]. Literature has identified some forms of green process technology, such as clean production, pollution control, pollution prevention, eco-efficiency, and recirculation [4,5,6,7]. As the government and the public pay more attention to environmental issues, GPI has become an important way for firms to maintain competitiveness [9,35]. Scholars generally believe that technology innovation needs multiple participants’ cooperation [5,44]. Compared with traditional innovation, GPI represents a technological frontier on which firms are inexperienced, and relies more on skills and knowledge across organizational boundaries [1]. This peculiarity makes routine repetition across firms possible [30].

Routine replication refers to the transfer of routine across firms [30]. Its essence is the transfer of knowledge sets [31,32,45] and it is the key component of organizational learning [46,47]. Two types of routine replication are often mentioned according to the knowledge transferred, zero-order routine replication (ZRR) and higher-order routine replication (HRR) [48,49]. The knowledge template involved in ZRR is clear and structurally stable [49]. Through the split and reconstruction of the knowledge template, existing knowledge could be improved. The knowledge contained in HRR is diversified, complex, and changeable [50,51]. HRR needs a firm break through existing partnerships and conducts extensive knowledge search. It can change or adapt the original knowledge template [49], so has the potential to enable firms to do something radically different [52].

### 2.2. The Impact of Environmental Management on Green Process Innovation

Prior research in the domain of innovation management has shown that technological innovation cannot be developed spontaneously [25]. It is heavily dependent on managerial practices [29]. We further introduce this standpoint to GPI.

EM could provide corresponding resources and support for GPI. First, firms that implement EM often set environmental goals in their organizational strategies. This allows top executives to incorporate environmental issues into their decisions, to consider environmental issues when allocating resources, and to provide funding, equipment, personnel, and other support for GPI [27,41]. Second, the adjustment of the organizational structure has made EM a coordination mechanism and cross-firm collaboration that seeks solutions [28,42,43]. Third, the systematic environment monitor and audit help firms to identify existing environmental problems, uncover potential opportunities, and induce GPI [3,36]. Finally, the continuous learning and employee training are conducive to the expansion of employee skills and knowledge, providing a knowledge base for the development of green processes [44]. Therefore, firms’ EM would stimulate and support GPI. This paper proposes the following hypothesis:

H1: EM has a positive effect on GPI.

### 2.3. The Impact of Environmental Management on Routine Replication

Although organizational routine is likened to genes by theorists [46,47], it is not necessarily unchanging [53]. Selective managerial practices are necessary to explain routine replication [50]. Firms with proactive environmental strategies collaborate intensively with their partner [1], which provides the foundation for routine replication across firms.

As a management system, EM focuses on environmental impacts, identifies environmental risks, and takes initiatives [29]. The setting of environmental goals, adjustment of organizational structures, and deployment of employees enable firms to face environmental problems and try to find solutions [14,53]. Although firms in the same industry adopt similar process technology, resulting in the same pollutants and emissions, there are no widespread accepted standards on either environmental solutions or environmental performance measures. In order to tackle the complex of environmental issues, a variety of specialist knowledge which spreads within different firms is essential [1]. So, collaboration and cooperation among industry peers are needed. Firms communicate and share their pollution abatement experience through collaboration, and knowledge transfer becomes possible [54,55]. Taking the Chinese manufacturing industry as an example, in order to dispose wastes and emissions in production process, firms often send employees to attend industry conferences. This measure enables firms to learn from peers, share their experiences, and brings in ZRR. Besides this, a strategic alliance launched by large proactive manufacturing companies and research institutions was setup. This enabled deep cooperation, extensive knowledge exploration, and HRR across firms to become possible. Therefore, this study proposes the following hypotheses:

H2a: EM has a positive effect on ZRR.

H2b: EM has a positive effect on HRR.

### 2.4. The Impact of Routine Replication on Green Process Innovation

Scholars use routine replication to illustrate the process of technology innovation [5,7,31]. We specifically focus on GPI. Routine replication provides the basis for the development of GPI. It is a dynamic learning process that realigns organizational knowledge templates [46,56]. It helps to break knowledge stickiness, reduce barriers in knowledge transfer, and accelerate the formation of new knowledge [46]. The improvement of organizational capability due to knowledge transfer is more likely to stimulate technology innovation [44].

The knowledge involved in ZRR is concrete and clear, and existing knowledge could be used in depth [32,57,58]. Due to the effect of ZRR, firms identify and perceive knowledge units in existing knowledge bases, and reorganize them. When this knowledge is applied to production, it will lead to the adaption of existing process, and the reduction of a firm’s pollution [59]. 

The knowledge involved in HRR is complex and implicit, which enables the creation of new knowledge [32,60]. HRR works when ZRR does not lead to satisfactory results [32,54]. It usually guides firms to deconstruct and reconfigure existing knowledge and form new knowledge [56]. Under the role of HRR, firms can communicate deeply and cooperate widely on generic environmental problems with their peers, laying a foundation for the introduction of novel production process. Taking the example above, after years of deep cooperation, a novel process technology was developed by the industry strategic alliance. The wastes and pollutants emitted during the production process were reduced sharply. Therefore, the following hypotheses are proposed:

H3a: ZRR has a positive effect on GPI.

H3b: HRR has a positive effect on GPI.

### 2.5. The Mediating Effects of Routine Replication

The above analysis shows that EM provides conditions for routine replication across firms, and routine replication is conducive to GPI. Therefore, routine replication plays a mediating role in the relationship between EM and GPI. The following hypotheses are proposed:

H4a: ZRR plays a mediating role in the relationship between EM and GPI.

H4b: HRR plays a mediating role in the relationship between EM and GPI.

Existing research on GPI shows that this type of innovation is a revision of traditional innovation and a leap in the original innovation trajectory, therefore has higher uncertainty [61,62]. Most firms still lack experience in this regard. In addition, the implementation of green technology is a quite complex task that often requires information and skills that are different from the industry’s traditional knowledge base. It requires firms to explore knowledge more broadly and effectively [62]. Compared with ZRR, which has a stable structure, HRR is more flexible [32,58]. By expanding external contacts and searching for knowledge broadly, it can break innovation barriers, disperse the risk of innovation, and increase the success of GPI. Therefore, this paper proposes the following hypothesis:

H4c: HRR has a stronger mediating effect in the EM–GPI relationship than ZRR.

ZRR is the exploitation and utilization of existing knowledge base [32,57,58]. Simply emphasizing ZRR will lead to rigidity of the firm, which is not conducive to getting rid of the traditional technology path. HRR emphasizes the expansion and exploration of knowledge base [32,57,58]. However, a simple emphasis on HRR without considering the existing competence of the firm will lead to high innovation cost and low innovation efficiency [63]. Taking into account the complementary characteristics of the two types of routine replication, the following hypothesis is further proposed:

H4d: There is a complementary relationship between ZRR and HRR, and the mediating effect of the interaction between these two types is stronger than that of an individual effect.

**Figure 1:**
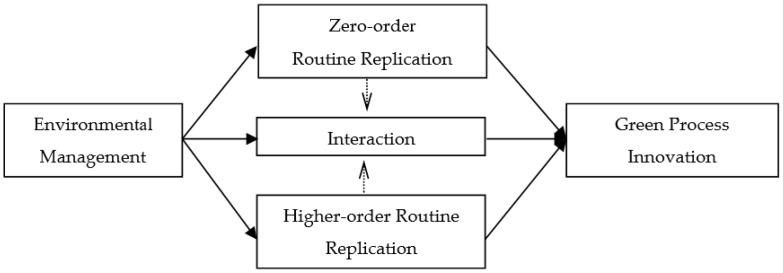
Theoretical framework.

The theoretical framework of this paper is shown in Figure 1.

## 3. Research design

### 3.1. Sample and Data Collection 

The manufacturing industry is not only a large consumer of energy and natural resources, but also a major emitter of pollutants, facing strong environmental pressures [64] and is often selected as a sample in environmental management research [19,33,39,40,55]. This study selects the Chinese manufacturing industry as a research sample.

Since one of the themes of this research is routine replication and there is a lack of such information in public data, the data were collected by means of questionnaire. The questionnaire was issued in Shandong Province, which is located on the eastern coast of China. In 2017, its GDP was in third place among the country’s provinces, accounting for 11% of the national total. As a polite province in China, it launched a strategy to switch old growth drivers to new ones in 2018. One of the aims of this strategy is to continuously improve environmental quality without affecting economic development. Since then, the provincial government has taken more efforts to promote environmental management. It is very suitable to select this province as the place of questionnaire distribution. The list of sample firms came from local enterprise association. Before the questionnaires were issued, we communicated with firms’ directors by telephone to clarify the purpose of the investigation and entrust them to find suitable personnel to finish the questionnaire. The informants were experienced technical supervisors, environmental supervisors, and senior managers in their firms. A total of 600 questionnaires were distributed and 161 questionnaires were collected after two months. For the unresponsive firms, we made a second telephone contact, after which 95 copies were received. In total, 225 valid questionnaires were obtained after removing invalid questionnaires. The demographic traits of the informants and firms can be seen in Appendix A.

In order to test whether non-respondent bias exists, we compared the first group of responding firms with the second group. The *t*-test results show that there are no significant differences between the two groups in terms of age, scale, and ownership. Therefore, non-respondent bias is excluded.

### 3.2. Variable Measurement

Most of the existing literature has adopted environmental management certification (ISO14001) as a research variable in the domain of EM. As the cost of environmental management certification is relatively high, only a few firms in developing countries have passed such certification. For most firms, they have adopted a more flexible and cost-effective voluntary environmental management [65]. We used voluntary environmental management as a research variable. The EM scale refers to the research of Kolk and Mauser [17] and Daddi et al. [39], and uses six items, such as organizational goals, organizational structure, and employee training. Routine replication is primarily based on the research of Winter and Szulanski [45], Gupta et al. [56], Pentland et al. [66], and Bresman [67]. ZRR was measured by the clear stability of acquired knowledge, while HRR was measured by the complex variability of acquired knowledge, and six items were adopted to conduct the measurements. GPI mainly refers to the research of Amores-Salvadó et al. [68], Bossle et al. [69], and Ma et al. [70], and was measured by three items. All scales adopted the five-point Likert scale, with 1 indicating strongly disagree and 5 indicating strongly agree. The informants were asked to recall their EM practices over the past 3 years and their process improvement during the last year [5,55] in order to avoid the potential simultaneous causality between EM and GPI. See Appendix B for specific items. 

Control variables were set as follows: Firm size (the logarithm of employee numbers), firm age (the logarithm of firm’s age), types of industry (take the manufacturing double-digit code issued by the China Securities Regulatory Commission), and ownership (state-owned = 1, non-state-owned = 0).

In addition, in order to avoid the social desirability bias and the common method bias, we disrupted the items’ order and added the reverse item in the questionnaire [71].

### 3.3. Method

Descriptive statistical analysis of the samples was performed by using SPSS 25.0 software (SPSS Inc., Chicago, IL, USA), the reliability and validity of each scale were tested, and correlation analysis and multiple regression analysis were performed as well. In addition, with the help of Lisrel 8.7 software (Scientific Software International, Inc., Philadelphia, PA, USA), the robustness of the above results was tested by the bootstrapping method and the mediating effects were compared.

## 4. Empirical Results and Discussion

### 4.1. Reliability and Validity

The Cronbach’s α coefficients of EM, routine replication, and GPI are 0.802, 0.829, and 0.924, respectively, showing that the reliability of the scale satisfies research needs [72]. 

An item in EM scale was eliminated after factor analysis, since it spanned two factors. After the elimination, the EM scale had five items. The corrected KMO coefficient is 0.726 (*p* = 0.000), and a common factor was extracted, which was the same as the initial construct. The KMO coefficient of the routine replication scale is 0.763 (*p* = 0.000). Two principal components were extracted, corresponding to ZRR and HRR, respectively, which are consistent with the initial construct. The KMO coefficient of GPI scale was 0.866 (*p* = 0.000). One principal component is extracted, which is consistent with the initial construct.

According to the Harman single factor test, the one with the largest load explains 19.8% of the variance variation, excluding the possibility of the common method bias [73].

### 4.2. Correlation Analysis

The correlation coefficients of the independent variable (EM), the dependent variable (GPI), and mediators (ZRR and HRR) are shown in Table 1. EM is significantly positively correlated with ZRR and HRR, and is also significantly positively correlated with GPI. ZRR and HRR are significantly positively correlated with GPI, laying the foundation for further analysis.

### 4.3. Regression Analysis

Considering the potential simultaneous causality between EM and GPI, the Hausman test was used and the result shows that the endogeneity problem does not exist [74]. Multiple regression analysis was used in the next steps. The results are shown in Table 2. The dependent variable in Model 1 is GPI. The coefficient of the independent variable (EM) is 0.609 (*p* < 0.001), indicating that EM contributes to GPI. Therefore, hypothesis 1 is supported. This result consolidates extant literature [22,29]. The dependent variables in Model 2 and Model 3 are ZRR and HRR, respectively. The coefficients of EM in these models are 0.517 (*p* < 0.001) and 0.453 (*p* < 0.001), respectively, indicating that EM contributes to both ZRR and HRR. Hypotheses H2a and H2b are supported. The coefficients of routine replication for GPI in Model 4 and Model 5 are 0.443 (*p* < 0.001) and 0.392 (*p* < 0.001), respectively, indicating both ZRR and HRR contribute to GPI. Therefore, hypotheses H3a and H3b are supported. Model 6 further examines the mediating effect of ZRR. It shows that comparing with Model 1, the coefficient of EM on GPI decreases, indicating that ZRR plays a partial mediating role. Hypothesis H4a is supported. EM and HRR were added in Model 7. The result indicates that a partial mediating effect exists. Hypothesis H4b is supported. This result is different from that of literature [5], which we will discuss in the next subsection. The mediating effect of the interaction wass tested by comparison between Model 8 and Model 1, also showing a partial mediating effect.

In order to compare the above three mediating effects, this study used the bootstrapping method by Mplus 7.0 software (sample size was set to 2000, and the confidence interval was set to 95%) [75]. The results are shown in Table 3. The confidence intervals corresponding to the aforementioned hypotheses (a1 corresponding to H2a, a2 corresponding to H2b, b1 corresponding to H3a, b2 corresponding to H3b, a1 × b1 corresponding to H4a, and a2 × b2 corresponding to H4b) do not include 0, indicating the robustness of these results. In addition, the confidence interval of a2 × b2 − a1 × b1 is (−0.009, 0.087), including 0, indicating that there is no significant difference in the mediating effect between ZRR and HRR. Therefore, hypothesis H4c is not supported. The confidence interval of a3 × b3 − a1 × b1 is (0.021, 0.032) and that of a3 × b3 − a2 × b2 is (0.025, 0.171). Both intervals are located on the right side of 0, indicating the mediating effect of the interaction is stronger than that of the individual, and hypothesis H4d is supported.

### 4.4. Discussion

Although some scholars doubt the effect of EM [33,34], our empirical research gives evidence that EM contributes to firms’ GPI. The implementation of EM could decompose the organization’s environmental objectives into middle-level departments and grass-root employees. Employee training and continuous learning could provide employees with appropriate skills. Employees focus on environmental issues and try to find solutions in their daily operation under the support of EM. The improvement and adaption of production processes become possible. 

Both ZRR and HRR have positive effects on GPI and play partial mediating roles in the EM–GPI relationship. This conclusion implies that the transition from traditional technology to green technology is an incremental process. The success of GPI is inseparable from the existing technology base [76]. Our result is different from that of Zhang and Zhou [5]. They only found a mediating role played by ZRR. We attribute this difference to the measure scale used. In their research, GPI was limited to the pollution control technique. This kind of technology has strong versatility and less interference to the existing production system [20]. ZRR is mainly related to the general operational capability of firms and is suitable for stable organizational environment [50]. So, ZRR is conductive in this context. While, in our research, the green process includes both pollution control and pollution prevention. Pollution prevention will bring some problems to the existing production system [20]. HRR is suitable for this kind of turbulent business environment [51]. 

The interaction between ZRR and HRR plays a stronger mediating effect. This conclusion supports the view of Raisch et al. [63]. Blind emphasis on HRR leads to a lack of solidity in the firm’s own capability, while blind emphasis on ZRR would make firms less self-sufficient and less adaptable [77]. The interaction of the two routine replications plays a stronger mediating role, indicating the complementary relationship between these two types of routine replication.

## 5. Conclusions and Implications

### 5.1. Conclusions

Through the above empirical tests, we can draw the following conclusions. First, EM positively relates to firms’ GPI. Second, routine replication plays a mediating role. Last but not least, the mediating effects played by ZRR and HRR are equally important and a balance between them is beneficial to the focal firm. 

### 5.2. Implications

Our conclusions can give implications to both academia and managerial practices.

To the best of our knowledge, there is little research interpreting the relationship between EM and GPI. Inoue et al. [3] posit that EM is beneficial to green innovation. De Marchi and Grandinetti [1] point out that firms with proactive environmental strategies incline to adopt broad external connections with peers and research institutions, which is a key enabling condition for routine replication [46]. Our research is a complementation and further development of the earlier research. We confirm that EM is conducive to GPI. When firms adopt systematic EM practices, they tend to communicate with industry peers and institutions, and try to improve or adapt process technology to solve the genetic environmental issues. 

As the focus of this research was on how to introduce GPI within firms, it can give some implications to business managers. Firms’ capability with regard to GPI depends on their environmental policies and practices. Firms’ EM practice is top-down systematic engineering, including upper-level environmental objectives, middle-level organizations, and the active participation of grassroots. All employees, both department managers and grassroots, should know their firm’s environmental responsibility and try to find solutions. Other managerial measures are conducive, e.g., employee training. The implementation of EM is helpful to obtain GPI. Practitioners should have confidence in it.

It is also helpful for firms to expand their business connections. GPI cannot be separated from the support of the industrial chain because of its complexity, externality, and uncertainty. An open mind is needed for practitioners. The more opportunities offered for employees to communicate with peers, research institutions, and suppliers, the more knowledge transfers and more routine replicates from outsides. So GPI can be developed.

Besides such managerial initiatives mentioned above, managers should focus on the balance between ZRR and HRR. Overemphasis on HRR will lead to the instability of firms or result in a “failure trap”. 

## 6. Future Research Directions

This study explores the impact of EM on GPI and its impact path. It shows that ZRR and HRR both play mediating roles in the influence of EM on GPI. Additionally, complementary effect exists between them. As firms’ operations are constrained by existing resource conditions, how to maintain a balance between ZRR and HRR needs to be addressed continuously in future research. 

## Figures and Tables

**Table 1:** Results of correlation analysis of variables.

Variables	Mean	S. D.	EM	ZRR	HRR
EM	3.783	0.895	1.000		
ZRR	3.989	0.679	0.261 **	1.000	
HRR	3.722	0.633	0.410 **	0.449 **	1.000
GPI	3.464	0.661	0.525 **	0.375 **	0.344 **

Note: ** *p* < 0.01. S.D. = standard deviation; EM = environmental management; ZRR = zero-order routine replication; HRR = higher-order routine replication; GPI = green process innovation.

**Table 2:** Results of regression analysis.

Variables	GPI	ZRR	HRR	GPI				
	Model 1	Model 2	Model 3	Model 4	Model 5	Model 6	Model 7	Model 8
SIZE	−0.089	−0.054	−0.087	−0.097	−0.056	−0.093	−0.097 *	−0.099 *
AGE	−0.124 **	−0.159 **	−0.100	−0.045	−0.075	−0.021	−0.117 *	−0.096 *
PRO	−0.126 **	−0.112 *	−0.116 **	−0.075	−0.111 *	−0.081 *	−0.115 *	−0.082
IND	0.018	0.146	0.185	−0.097 *	−0.107 *	−0.040	0.001	−0.039
EM	0.609 ***	0.517 ***	0.453 ***			0.404 ***	0.437 ***	0.376 ***
ZRR				0.443 ***		0.396 ***		
HRR					0.392 ***		0.379 ***	
INTER								0.541 ***
R^2^	0.409	0.391	0.359	0.407	0.345	0.505	0.490	0.592
Adjusted R^2^	0.403	0.384	0.352	0.400	0.335	0.497	0.472	0.583
F	60.086	55.671	48.574	59.389	54.682	66.010	64.994	70.588
	***	***	***	***	***	***	***	***

Note: * *p* < 0.05, ** *p* < 0.01, *** *p* < 0.001, data in this table are standardized coefficients. EM = environmental management, GPI = green process innovation, ZRR = zero-order routine replication, HRR = higher-order routine replication, INTER = zero-order routine replication × higher-order routine replication, SIZE = firm size, AGE = firm age, OWN = ownership, IND = industry.

**Table 3:** Results of bootstrapping analysis.

Coefficient	Estimated Value	Standard Error	95% Confidence Interval		*p*
			Lower	Upper	
EM→ZRR (a1)	0.356	0.081	0.123	0.589	0.004
EM→HRR (a2)	0.311	0.129	0.060	0.557	0.016
EM→INTER (a3)	0.470	0.078	0.298	0.642	0.028
ZRR→GPI (b1)	0.345	0.059	0.161	0.529	0.000
HRR→GPI (b2)	0.270	0.031	0.178	0.362	0.003
INTER→GPI (b3)	0.387	0.091	0.137	0.636	0.006
EM→ZRR→GPI (a1 × b1)	0.123	0.021	0.067	0.179	0.008
EM→HRR→GPI (a2 × b2)	0.084	0.017	0.049	0.119	0.036
EM→INTER→GPI (a3 × b3)	0.182	0.052	0.051	0.313	0.012
a2 × b2 − a1 × b1	−0.039	0.022	−0.009	0.087	0.031
a3 × b3 − a1 × b1	0.059	0.021	0.032	0.115	0.008
a3 × b3 − a2 × b2	0.098	0.028	0.025	0.171	0.009

Note: EM = environmental management, ZRR = zero-order routine replication, HRR = higher-order routine replication, INTER = interaction, GPI = green process innovation.

## Appendix A

**Table A1:** **Demographic traits of informants and sample firms**.

Informants		Sample firms	
Department		Ownership	
Environmental protection	24%	State-owned	39.5%
Technical	23.1%	Non-state-owned	60.5%
Others	45.3%	Age	
Position		No more than 5 years	14.2%
Junior manager	31.1%	5 to 10 years	28.9%
Senior manager	45.3%	More than 10 years	56.9%
Top manager	23.6%	Industry	
Work experience within the firm		Petrochemical	3.6%
No more than 5 years	34.2%	Machinery and equipment	9.7%
5 to 10 years	21.8%	Pharmaceutical products	16.4%
More than 10 years	44%	Clothing	8.9%
		Electronics manufacturing	6.2%
		Food and beverage	23.1%
		Paper and printing	5.7%
		Rubber products	12.9%
		Others	13.5%

## Appendix B

**Table A2:** **Survey items**.

Please rate the level of your firm’s involvement in each of the following practices during last year. (1 = very low; 5 = very high)		
GPI	A1 The emission of hazardous substances is reduced in the production process.	Amores-Salvadó et al., 2014; Bossle et al., 2016; Ma et al., 2017
	A2 The energy efficiency is increased in the production process.	
	A3 The material efficiency is increased in the production process.	
Please rate the level of your firm's involvement in each of the following practices during the past 3 years. (1 = very low; 5 = very high)		
EM	B1 I'm not sure which department is responsible for the environmental management of our company. (Reverse)	Kolk and Mauser, 2001; Daddit et al., 2016
	B2 Firm leaders encourage to consider the environmental sustainability and green values as a shared vision for the organization.	
	B3 Our company has hardly carried out relevant employees trainings in view of environmental problems. (Reverse)	
	B4 Firms have clear environmental goals.	
	B5 Our company often publishes documents related to environmental management. (Deleted)	
	B6 Our company regularly evaluates the environmental performance.	
Routine Replication	C1 Firms have obtained clear and stable technical knowledge, market knowledge and cooperation specifications from their partners.	Winter and Szulanski, 2001; Pentland et al., 2012; Bresman, 2013; Gupta et al., 2015
	C2 Firms tend to maintain existing partnerships and strengthen knowledge transfer channels.	
	C3 Firms are able to adopt exploitative learning to implement and evaluate norms on time.	
	C4 Firms have acquired complex and varied technical knowledge, market knowledge and cooperation specifications from their partners.	
	C5 Firms tend to expand their scopes of cooperation to establish knowledge transfer channels.	
	C6 Firms are able to adopt exploratory learning to receive and change organizational norms in a timely manner.	
